# Epigenetics of hepatocellular carcinoma

**DOI:** 10.1186/s40169-019-0230-0

**Published:** 2019-05-06

**Authors:** Tan Boon Toh, Jhin Jieh Lim, Edward Kai-Hua Chow

**Affiliations:** 1 Cancer Science Institute of Singapore, National University of Singapore, 14 Medical Drive, MD6 #12-01, Singapore, 117599 Singapore; 2 Department of Pharmacology, Yong Loo Lin School of Medicine, National University of Singapore, 10 Medical Drive, Level 5, Singapore, 117597 Singapore

**Keywords:** Epigenome, Liver cancer, HCC, DNA methylation, Histone methylation, ncRNA

## Abstract

In recent years, large scale genomics and genome-wide studies using comprehensive genomic tools have reshaped our understanding of cancer evolution and heterogeneity. Hepatocellular carcinoma, being one of the most deadly cancers in the world has been well established as a disease of the genome that harbours a multitude of genetic and epigenetic aberrations during the process of liver carcinogenesis. As such, in depth understanding of the cancer epigenetics in cancer specimens and biopsy can be useful in clinical settings for molecular subclassification, prognosis, and prediction of therapeutic responses. In this review, we present a concise discussion on recent progress in the field of liver cancer epigenetics and some of the current works that contribute to the progress of liver cancer therapeutics.

## Liver cancer—hepatocellular carcinoma (HCC)

Liver cancer is the second most lethal cancer worldwide [1]. Liver cancer presents an important public health issue in many countries due to its highly aggressive nature and poor survival rate. Hepatocellular carcinoma (HCC) is the most prevalent form of the primary liver cancer and accounts for up to 90% of all cases. The incidence rates for HCC is rising in many countries due to increasing associated risks factors such as diabetes and obesity [2]. Other well-recognized risk factors for HCC includes chronic hepatitis B or C infection, exposure to dietary aflatoxin, alcohol-induced cirrhosis and smoking.

Deregulation of gene expression and aberrant molecular signalling confer survival advantages to cancer cells and are key hallmarks of cancer. Recently, it has been shown that undesirable changes in epigenetic alterations may enhance the selective advantage of cancer cells [3]. The reason why it is important to study epigenetics in the liver is due to the fact that it is the one of the organs that is constantly adapting to highly variable environmental conditions. The liver constantly adapts to circadian cues, metabolic processes, changes in the microbiota, and external factors such as viral infections and xenobiotics which results in the need for its constant repair and regeneration [4]. Therefore, the liver epigenome is extremely sensitive to its highly variable environment. As such, metabolic risk factors such as obesity, excessive alcohol consumption and insults from viral hepatitis cause a disturbance in the hepatic epigenome. Alterations of the epigenome such as DNA methylation, chromatin modification, miRNAs, and lncRNAs propels uncontrolled cell growth and proliferation, invasion and metastasis as well as the progression of liver cancer from chronic inflammation, fibrosis, subsequent accumulation of mutations and consequently, liver cancer [4–6].

With the advancement of next generation DNA sequencing, our understanding of the genetic and molecular pathobiology of liver cancer has increased dramatically. Large international efforts have been initiated to provide researchers with comprehensive genomic/epigenomic data publicly. Two of the well-known large public cancer genomic databases include the international cancer genome consortium (ICGC; http://www.icgc.org) and the cancer genome atlas (TCGA; https://cancergenome.nih.gov/). Both public databases aim to generate a comprehensive information of genomic abnormalities in cancer such as somatic mutations, aberration expression of genes and epigenetic modifications that will be made publicly available to the research community. Another initiative launched in 2010 is the international human epigenome consortium (IHEC; http://ihec-epigenomes.org) with the goal of generating comprehensive reference maps of human epigenomes for key cellular states such as stemness, differentiation, proliferation, stress, senescence, and others relevant to human health and disease. With these multiple platforms of public databases and others, the understanding of epigenetic programming implicated in cancers and the prevention and treatment of these cancers will be greatly improved.

## DNA methylation and HCC

**Fig. 1:**
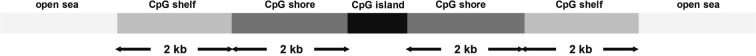
Schematic diagram displaying CpG annotations of genomic regions

DNA methylation is a process whereby DNA methyltransferases (DNMTs) transfer methyl groups from *S*-adenosyl methionine to cytosine bases of CpG dinucleotides at gene promoters and regulatory regions [7]. DNA methylation commonly occurs at the CpG dinucleotides in somatic cells with about 25% occurring in a non-CpG manner in embryonic stem cells (ESCs). CpG dinucleotides are commonly found in “CpG islands”, which are short CpG-rich regions. CpG islands, which occupy more than 50% of all promoters, can be methylated during development and promotes long-term gene silencing such as in the case of X-chromosomal inactivation and the imprinted genes. CpG shores are defined commonly as regions of low CpG density that are located within 2 kb up- and downstream of a CpG island (Fig. 1). CpG shelves refer to a region 2 kb outside of CpG shores, while regions with low methylation and are uncharacterized are known as CpG oceans. DNA methylation is tightly regulated by a family of DNMTs that consists of DNMT1, DNMT2, DNMT3A, DNMT3B and DNMT3L [8, 9]. DNMT1 has been proposed to be the maintenance methyltransferase that preferentially methylates hemi-methylated DNA over non-methylated DNA to maintain the original DNA methylation pattern during replication [10, 11]. DNMT3A and DNMT3B, on the other hand, are more likely to perform de novo methylation on unmethylated CpG dinucleotides during the developmental process [12]. In addition, cooperation of several DNMTs are also required to methylate certain regions of the genome, specifically the repetitive elements.

**Fig. 2:**
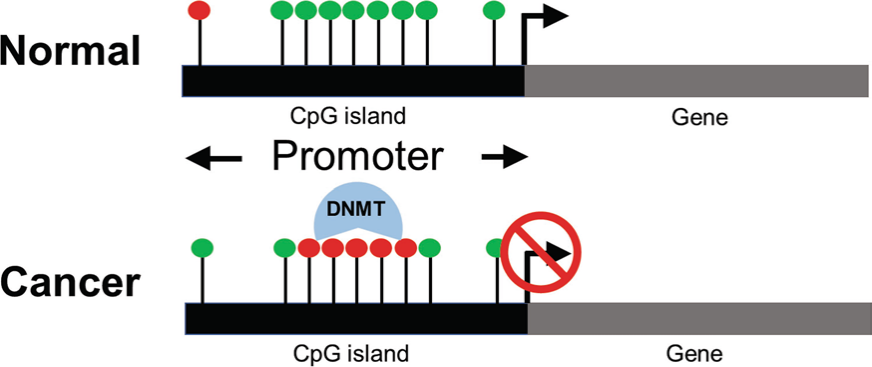
DNA methylation profile in cancer. Liver cancer cells typically exhibit DNA hypermethylation at promoter sites of tumor suppressor genes, resulting in silencing of these tumor suppressive genes

Dysregulated DNA methylation is commonly observed in many cancers including HCC [13]. The earliest indications that provide a link between epigenetics and cancer came from studies that correlate gene expression data and DNA methylation. Epigenetic changes such as global hypomethylation and specific gene promoter hypermethylation (Fig. 2) have been demonstrated to be involved in genome instability and tumor suppressor gene silencing respectively [14]. In HCC, dysregulated DNA methylation is one of the early events in HCC pathogenesis and plays an important role in elevating chromosomal instability [15]. Table 1 summarizes a list of DNA methylation studies in HCC.

**Table 1:** DNA methylation and HCC

Study objectives	Methods	Type of sample	Genes identified		Key findings	Implications of study	Refs.
			Hypo-methylated	Hyper-methylated			
To investigate the global methylation profile of purified single hepatocytes	Illumina Infinium Human Methylation27 BeadChip, COBRA and bisulfite sequencing	Single hepatocytes isolated from HBV-positive HCC (HBHC) tissues	–	*EMILIN2, WNK2, TM6SF1, TLX3, HIST1H4F, TRIM58, GRASP*	Hepatocyte methylation profiles can be classified into 3 groups based on hepatocyte origin: HCC, adjacent tissue and normal liver. 7 novel genes were found to be aberrantly methylated in HBHC	These genes can be potential novel biomarkers of HBHC once validated in larger clinical cohorts	Tao et al. [16]
To identify genes hypermethylated in HCC that can be detected in plasma DNA for early diagnosis	Illumina Infinium Human Methylation27 BeadChip	62 paired HCC tumor and NAT	*CCL20, AKT3, SCGB1D1, WFDC6, PAX4, GCET2, CD300E, CD1B, FLJ00060, MNDA*	*DAB2IP, BMP4, ZFP41, SPDY1, CDKN2A, TSPYL5, CDKL2, ZNF154, ZNF540, CCDC37*	684 CpG sites significantly hypermethylated in HCC tissues. 5 of these genes (*CDKL2, CDKN2A, HIST1H3G, STEAP4, ZNF154*) had detectable hypermethylated DNA in plasma of up to 63% of patients	Measuring DNA methylation from patient plasma is feasible. Panel of methylated genes identified can be potential biomarkers for early diagnosis	Shen et al. [17]
To study aberrant DNA methylation in HCC using higher resolution genome-wide analysis	Illumina HumanMethylation 450 BeadChip	27 HCC and 20 NAT	NFATC1	BMP4, CDKN2A, GSTP1	Greater global hypomethylation patterns observed in HCC compared to NAT, with higher frequency of events occurring in promoter CpG islands than CpG shores and shelves	Allows deeper understanding of differential methylation patterns in various gene regulatory regions	Song et al. [18]
To identify tumor suppressor genes silenced by DNA methylation in HCC	Illumina Infinium Human Methylation27 BeadChip, combined with microarray analysis of gene re-expression studies	71 primary HCC tissues, 8 non-diseased normal tissues, 4 HCC cell lines	–	ACTL6B, C19orf30, DGKI, DLX1, ELOVL4, LDHB, LRAT, MLF1, NEFH, PPM1 N, PRPH, SLC8A2, SMPD3	Identified 13 candidate tumor suppressor genes; *NEFH* and *SMPD3* were functionally validated in vitro and in vivo. Low levels of SMPD3 were associated with early HCC recurrence after curative surgery in an independent patient cohort	SMPD3 identified to be a potent tumor suppressor gene and could be an independent prognostic factor for early recurrence of HCC	Revill et al. [19]
To investigate novel genome-wide aberrant DNA methylation patterns in HCV-related HCC	Illumina Infinium HumanMethylation 450 BeadChip	66 pairs of HCC tumor and NAT			Identified 500 significant differentially methylated CpG sites that can distinguish HCC from NAT. Within NAT tissues, 228 CpG sites were identified to be significantly associated with HCV infection	Further functional studies would help to identify markers among the large subset of CpG sites/genes found to correlate with HCV infection, liver cirrhosis or HCC to aid in diagnosis and treatment	Shen et al. [20]
To investigate the genome-wide DNA methylation profile and identify stochastic epigenetic mutations (SEMs) in HCC	Illumina Infinium HumanMethylation 450 BeadChip	69 pairs of HCC tumor and NAT	*AJAP1, ADARB2, PTPRN2, SDK1* (hypermethylated at promoter level with concomitant hypomethylation at gene body level)		HCC tissues showed increased number of SEMs as compared to NAT. From a subset of SEMs unique to tumor tissues, 4 epigenetically-regulated genes that could be involved in HCC tumorigenesis were identified	Methylation and SEM profiles of HCC and adjacent non-cancerous liver tissues are highly different, allowing for the identification of important driver epimutations in HCC	Gentilini et al. [22]
To examine the effects of epigenetic alterations and features on the HCC genome architecture	Whole-genome bisulfite, whole-genome shotgun, long read and virus-capture sequencing approaches	373 HCC cases	NA		Somatic mutations occur preferentially in both highly methylated as well as hypomethylated regions in the liver cancer genome. HBV integration sites occur more frequently in inactive chromatin regions	Epigenetic features greatly influence the mutational processes in HCC. Understanding the mechanisms behind the interdependency between genetic, viral and epigenetic alterations in HCC can help in identifying epigenetic driver events	Hama et al. [23]

*COBRA* combined bisulfite restriction analysis, *HBV* Hepatitis B virus, *HCV* hepatitis C virus, *NAT* normal adjacent tissue

Aberrant hypermethylation of genes associated with HCC progression has been identified via several sequencing techniques. In an earlier study, Tao et al. performed a global methylation profile of single hepatocyte cells derived from hepatitis B positive HCC (HBHC) samples using Illumina Infinium Human Methylation27 BeadChips with combined bisulfite restriction analysis (COBRA) and bisulfite sequencing [16]. They found seven novel genes (*EMILIN2*, *WNK2*, *TM6SF1*, *TLX3*, *HIST1H4F, TRIM58* and *GRASP*) that were significantly methylated in HBHC but were hypomethylated in their respective paired adjacent tissues. These novel aberrant methylated genes could potentially be novel biomarkers for HCC once validated in larger clinical cohorts. In another similar study on predominantly HBHC samples, differentially methylated genes were identified using the Illumina Infinium Human Methylation27 in 62 paired HCC tumors and their adjacent non-tumor tissues [17]. Shen et al. demonstrated that the panel of methylated genes identified in HCC can be used as potential HCC-specific biomarkers of plasma DNA for early diagnosis of HCC. They showed that DNA methylation measurement in HCC patients’ plasma is feasible, with at least one of the genes from the panel being hypermethylated in 87% of the cases, thus supporting the utility of this panel of methylated genes as early biomarkers of HCC.

In a larger genome-wide methylation study conducted by Song et al. using the Methylation450 BeadChip, significant differential DNA methylation patterns in the CpG islands were observed in HCC as compared to their normal adjacent tissues [18]. Specifically, they found that global hypomethylation was observed in HCC and promoter CpG islands exhibited higher frequency of hypermethylation events than the regions surrounding the CpG islands, i.e. the CpG shores and the CpG shelves. To identify tumor suppressor genes in HCC, Revill et al. conducted a genome-wide methylation analysis of 71 human HCC specimens with microarray data analysis of gene re-expression in four HCC cancer cell lines and performed epigenetic unmasking by exposing the cells to reagents that induced reverse DNA methylation [19]. The authors identified 13 tumor suppressor genes, of which two (neurofilament heavy polypeptide, *NEFH* and sphingomyelin phosphodiesterase 3, *SMPD3*) were functionally validated in vivo.

A study by Shen et al. focused on the genome-wide DNA methylation profiles of hepatitis C virus (HCV)-related HCC tumors using the Infinium HumanMethylation 450K BeadChip arrays [20]. Consistent with previous findings, they observed higher percentage (79%) of hypomethylated CpG sites than hypermethylated sites (21%). In addition, hypermethylated CpG sites were more commonly found at the CpG islands and shores in contrast to the hypomethylated CpG sites that occurred mainly in the open sea region. More importantly, the authors were able to identify 228 aberrantly methylated CpG sites covering a total of 147 genes that had strong associations with HCV infection. Interestingly, there were no overlapping CpG sites in both HCV and HCC, signifying that HCV-associated methylated CpG sites are independent to HCC development. In another genome-wide DNA methylation profile study of 69 paired HCC tumor and adjacent normal liver tissues, Gentilini et al. used a different approach to identify epigenetic markers using epigenetic mutation analysis [21, 22] instead of using p-value or effect size [17, 18]. A gradual increase in the number of stochastic epigenetic mutations (SEMs) from normal liver, peritumoral tissues to HCC tissues was observed, with HCC tissues having 13-folds higher median SEMs than normal liver tissues. In addition, a list of novel potential epidrivers were identified by analysing genomic position of SEMs in both HCC and peritumoral tissues. These epigenes include *AJAP1*, *ADARB2*, *PTPRN2*, and *SDK1*.

A recent large scale epigenomic landscape study on 373 liver cancer specimens reported the correlation between epigenetic features and genetic aberrations using whole-genome bisulfite, whole-genome shotgun, long read and virus-capture sequencing [23]. Using a comprehensive genome and methylome sequencing approach, the authors identified two epigenetically distinct genomic regions in which somatic genetic aberrations were enriched—a region that is tumor specific hypomethylated and displayed an inactive chromatin genome, and the other region is an actively transcribing region with a highly methylated gene body area that is vulnerable to genetic insults and in part positively selected during carcinogenesis. In addition, this study also assessed whether the methylation status is associated with the distribution of somatic mutations by examining the correlation between somatic mutation density and methylation level. They showed that somatic mutations may occur preferentially in highly methylated regions of the non-cancerous liver genome, indicating that chromatin status may regulate the frequency of somatic mutations in liver cancer genome. Their integrative analysis provided evidence of interdependency between genetic, viral, and epigenetic alterations in liver cancer.

## ncRNAs and HCC

Noncoding RNAs (ncRNAs) can be categorized into two main subgroups according to their lengths [24]. Small or short noncoding RNAs include endogenous siRNAs and miRNAs that are less than 200 nucleotides. Long noncoding RNAs (lncRNAs) usually refer to RNAs more than 200 nucleotides in length. MicroRNAs (miRNAs), on the other hand, are small, noncoding RNAs of 18 to 25 bases in length that regulate post-transcriptional gene expression. In addition, the human genome has been reported to encode more than 1000 different miRNAs, each with distinct mRNA target(s). Hence, miRNAs represent a group of important epigenetic regulators that influence biological responses.

**Fig. 3:**
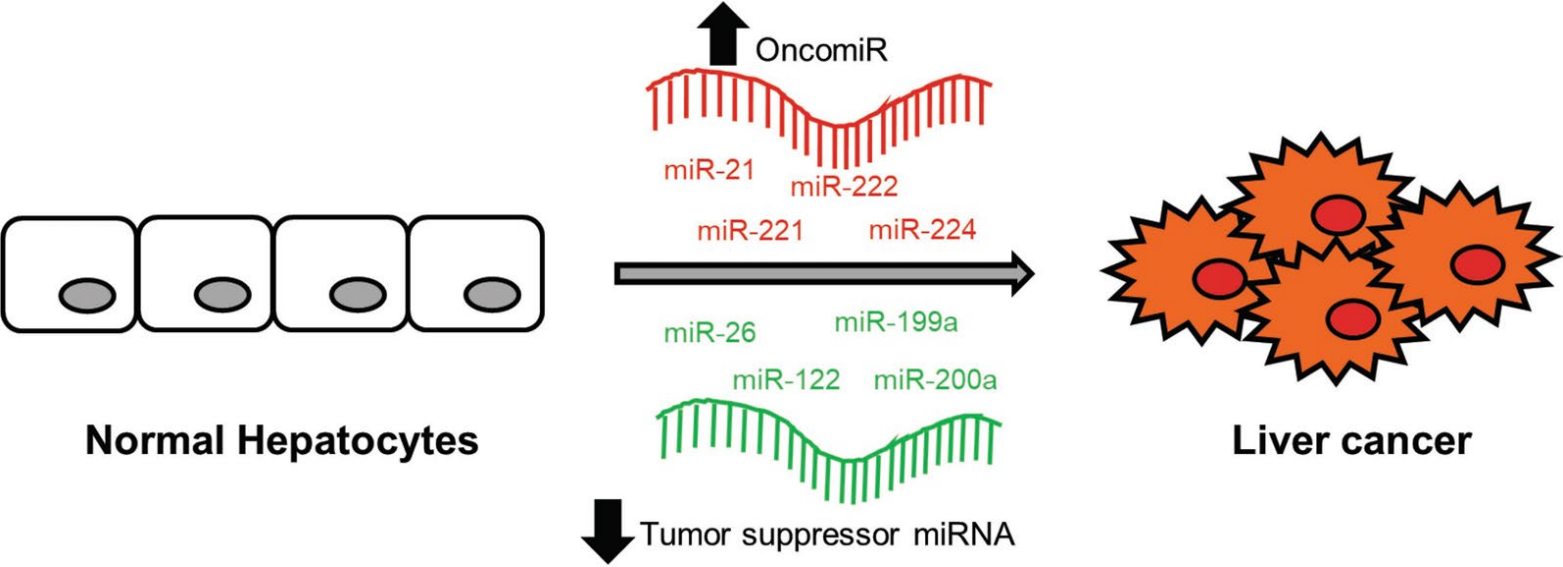
MicroRNAs in cancer. Elevation of oncogenic miRNAs (oncomiRs) results in silencing of tumor suppressor genes while downregulation of tumor suppressor miRNAs leads to reduced inhibition of oncogenes, consequently lead to the development of liver cancer

miRNAs are by far the most well-studied class of epigenetic regulators in liver cancer (Table 2). The first report of miRNA dysregulation in liver cancer is from Murakami et al. who reported the abnormal expression pattern of four miRNAs to be associated with HCC differentiation, namely miR-20, miR-92, miR-18 and precursor miR-18 [25]. Subsequently, numerous reports on miRNA dysregulation have been reported in HCC. Some of the consistently reported miRNAs that are differentially expressed in HCC tumors compared to normal liver tissues are miR-21, miR-26, miR-122, miR-199a, miR-200a, miR-221, miR-222, and miR-224 (Fig. 3).

**Table 2:** ncRNAs and HCC

ncRNA	Dysregulation in HCC	Role(s) in HCC	References
miRNAs			
miR-21	Upregulated expression	Directly targets and represses MAP2K3 expression Inhibition of miR-21 increased PTEN levels, affecting tumor cell proliferation and migration	[26, 27]
miR-221/222	Upregulated expression	Increased levels are associated with PTEN and TIMP3 downregulation Targets p27 and induces tumor proliferation	[28–30]
miR-224	Upregulated expression	Promotes tumor cell proliferation, migration, invasion; inhibits apoptosis Early HCC patients showed increased levels of serum miR-224; potential serum biomarker for early HCC detection	[31–34]
miR-26	Downregulated expression	Represses CDK6 and cyclin E1 expression, inhibiting G1/S transition Inhibits expression of VEGFA, suppressing angiogenic and proliferative ability of the tumor	[35–37]
miR-122	Downregulated expression	Marker for hepatocyte-specific differentiation Inhibits tumorigenic abilities of HCC cells Loss of miR-122 enhances tumor cell migration and invasion	[38–44]
miR-199	Downregulated expression	Low levels correlate with poorer survival Targets tumor-promoting PAK4, repressing the PAK4/Raf/MEK/ERK pathway	[45–47]
miR-200a	Downregulated expression	Inhibits cell growth, migration, invasion and EMT by targeting Foxa2 and ZEB2	[48, 49]
lncRNAs			
*HULC*	Upregulated expression	Associated with tumor proliferation, angiogenesis and metastasis Downregulates p18 tumor suppressor Acts as miRNA sponge to sequester tumor-suppressive miRNAs, which could lead to activation of EMT and tumor angiogenesis	[50–54]
*HOTAIR*	Upregulated expression	Recruits PRC2 and LSD1 complexes to mediate specific gene silencing Involved in maintaining HCC tumor microenvironment via CCL2 expression Inhibiting HOTAIR can suppress HCC proliferation and sensitise tumor cells to chemotherapy	[55–59]

Oncogenic miRNA that drives progression of HCC such as miR-21, miR-221, miR-222 and miR-224 are frequently found to be upregulated in HCC. For instance, miR-21 was found to be upregulated in HCC and inhibition of miR-21 resulted in a marked elevated expression of the tumor suppressor phosphatase and tensin homolog (PTEN) with accompanied reduction in tumor cell proliferation, migratory and invasive ability [26]. Furthermore, mitogen-activated protein kinase-kinase 3 (MAP2K3) was observed to be a direct target of miR-21 whereby MAP2K3 expression, which was repressed in HCC tissues, was observed to be inversely correlated with miR-21 [27]. miR-221 and miR-222 have been shown to be overexpressed in HCC and the elevated levels of these two miRNAs are correlated with *PTEN* and *TIMP3* downregulation [28]. In addition, Pineau et al. showed that miR-221/222 upregulation is an early event and have the highest elevated expression in HCC samples. It has been shown to target CDK inhibitor p27 to induce tumor proliferation and its overexpression is correlated with poorer prognosis [29, 30]. miR-224 is another commonly upregulated HCC-specific miRNA. miRNA-224 has been shown to promote proliferation, inhibit apoptosis, migration and invasion of HCC tumor cells [31, 32]. More importantly, miRNA overexpression has been found to correlate with poorer survival in HCC patients [33]. Furthermore, early HCC patients showed upregulated levels of serum miR-224 as compared to those with liver cirrhosis, chronic hepatitis B and healthy control subjects, highlighting the potential of miR-224 as a reliable serum biomarker for early HCC detection [34].

Tumor suppressive miRNAs are usually silenced in human liver cancers and these include miR-26, miR-122, miR-199a and miR-200a. miR-26 has been shown to be downregulated in HCC and could directly repress the expression of CDK6 and cyclin E1, which induced a decreased in the phosphorylation of retinoblastoma protein (pRb) [35]. More recently, miR-26 was observed to play a crucial role in tumor angiogenesis [36]. Specifically, downregulation of miR-26 correlated with enhanced angiogenic potential of HCC and gain-of-function studies showed that miR-26 was able to inhibit expression of vascular endothelial growth factor A (VEGFA) which subsequently suppressed tumor promoting properties of HCC cells such as proliferation, migration and in vivo tumor angiogenesis. miR-122 is the most abundant miRNA that accounts for 70% of the total miRNA population in the liver [37]. miR-122 expression has been frequently found to be repressed in HCC [38, 39] and is an important marker for hepatocyte-specific differentiation [40, 41]. Importantly, reduced miR-122 expression is correlated to a subset of HCC tumors with bad prognosis [42]. In addition, loss of miR-122 resulted in increased cell migratory and invasive phenotype. Since miR-122 correlates with several clinical parameters such as tumor size and invasiveness, it presents an attractive therapeutic target for HCC intervention [42–44]. miR199 is another abundantly expressed miRNA in the normal liver tissue that is downregulated in HCC. In particular, miR-199a-3p and miR-199a-5p are frequently found to be repressed in human HCC tissues [45, 46]. Furthermore, low miR-199a-3p expression is observed to be strongly correlated with reduced survival of HCC patients [47]. miR-199a/b-3p has been found to target the HCC tumor-promoting PAK4 by repression of PAK4/Raf/MEK/ERK pathway both in vitro and in vivo, suggesting that miR-199a/b-3p could be a potential therapeutic option for HCC [47]. Chen et al. reported that the decreased miR-200a expression in HCC could lead to abnormal cell growth, migration and invasion via the regulation of its target, transcription factor forkhead box A2 (Foxa2) [48]. Furthermore, lower miR-200a expression also enhanced the side population (SP) of HCC tumors to metastasize via transactivation of ZEB2 expression and the subsequent epithelial-mesenchymal-transition (EMT) activation in HCC tumor cells [49]. The major challenge for the use of miRNAs will be the targeted delivery and control of expression of these therapeutic miRNAs. With greater understanding of the roles and biology of miRNAs and the integration of current emerging delivery advances, miRNAs could represent an attractive alternative therapeutic treatment for HCC.

lncRNAs are also important modulators of HCC progression (Table 2). Due to the advances in genomic techniques, the role of lncRNAs as central regulators in genome regulation and dynamics has begun to emerge. Some of the known lncRNAs implicated in HCC are *HULC*, *HOTAIR*, *MEG3* and *HOTTIP*. *HULC* (highly up-regulated in liver cancer) was described by Panzitt et al. using HCC specific gene libraries and cDNA microarrays [50]. *HULC* is an oncogenic lncRNA approximately 1.6 kb in length and is highly upregulated in human HCCs. The upregulation of *HULC* is associated with tumor proliferation and is effected via downregulation of the p18 tumor suppressor. Additionally, the upregulation and stability of *HULC* is enhanced post-transcriptionally by IGF2 mRNA-binding protein 1 (IGF2BP1) [51]. Importantly, it has been shown that *HULC* is the first substrate to be destabilized by IGF2BP1, with CNOT1 protein as a crucial interacting partner. *HULC* is also known to act as a sponge to sequester miRNAs. Wang et al. showed that *HULC* inhibits and downregulates miR-372, leading to reduced translational repression of its target gene *PRKACB*, which is able to phosphorylate and activate cAMP response element binding protein (CREB), a key transcription factor in promoting proliferation and cellular adaptive responses [52]. Therefore, upregulation of *HULC* can lead to HCC progression by indirect activation of CREB transcription factor. Other miRNAs such as miR-107 and miR-200a-3p have also been shown to be sequestered by *HULC* and could subsequently lead to angiogenesis and EMT activation in HCC [53, 54]. *HOTAIR* (*ho*x *t*ranscript *a*ntisense *i*ntergenic *R*NA) is an intergenic lncRNA of approximately 2.2 kb in length that has been implicated in multiple solid cancers such as breast, colorectal, pancreatic and HCC. In HCC, *HOTAIR* has been demonstrated to be overexpressed and is able to interact and recruit polycomb group complex 2 (PRC2) and lysine specific demethylase 1 (LSD1) complexes to mediate specific gene silencing via histone 3 lysine 27 trimethylation (H3K27me3) repressive marks [55, 56]. Depletion of *HOTAIR* has also been shown to reduce HCC proliferation, suggesting its role in promoting tumor cell growth [57]. More importantly, suppression of *HOTAIR* in HCC cells sensitizes them to chemotherapeutic treatments such as doxorubicin and cisplatin, suggesting *HOTAIR* to be a biomarker of HCC tumor recurrence [58]. More recently, *HOTAIR* has been implicated in maintaining HCC tumor microenvironment via *HOTAIR*-induced C–C motif chemokine ligand 2 (CCL2) expression [59]. It was shown that CCL2 is a downstream target of *HOTAIR* and is involved in the recruitment of myeloid-derived suppressor cells (MDSCs) and macrophages to the tumor microenvironment.

## Chromatin modifiers and HCC

Chromatin modifiers or remodelers are an important class of proteins that take part in the regulation of accessibility to chromatin and positioning of nucleosome in the DNA [60]. Some of the well-studied chromatin modifiers in HCC include enhancer of zeste homologue 2 (EZH2), AT-Rich Interaction Domain 1A (ARID1A) and AT-rich interactive domain 2 (ARID2). EZH2, a methyltransferase belonging to the Polycomb Repressive Complex 2 (PRC2) which mediates gene silencing via H3K27me3, is frequently upregulated in HCC. It has been shown that depletion of EZH2 in HCC cells effectively reduced growth of HCC tumors and tumorigenicity in vivo [61]. More importantly, high expression levels of EZH2 is strongly associated with increased aggressiveness and metastatic properties along with poorer prognosis in HCC patients. In addition, EZH2 overexpression repressed miR-622 by enhanced H3K27 trimethylation, and is correlated with upregulation of CXCR4 and unfavourable prognosis in HCC patients [62]. More recently, EZH2 inhibition was found to facilitate natural killer (NK) cell-mediated cancer cell eradication through re-expression of NK cell ligands in HCC cells, suggesting the use of EZH2 inhibitors in rendering HCC cells more susceptible to NK-mediated cytotoxicity [63].

ARID1A and ARID2 are frequently found to be mutated in a subset of HCC patients [64, 65]. Both proteins belong to the SWItch/Sucrose Non-Fermentable (SWI/SNF) chromatin remodeling complexes and aid in regulating the accessibility of promoters to the transcriptional machinery. ARID1A encodes for BAF250a subunit of the SWI/SNF complex and has been shown to be a *bona fide* tumor suppressor based on several mutational and functional studies [66]. However, a recent study by Sun et al. described ARID1A as having both oncogenic and tumor suppressive roles that were context-dependent in HCC development and metastasis [67]. Specifically, the authors showed that while ARID1A supports initial HCC development, ARID1A loss after tumor establishment further accelerates and increases metastatic potential of HCC, suggesting the importance of protein subunit dosage in the proper regulation of global transcription. ARID2 belongs to the polybromo-associated BRG1-associated factor (PBAF) complex that can activate ligand-mediated transcription via nuclear receptors. ARID2 knockout studies showed that ARID2 is required for proper nucleotide excision repair (NER) of DNA damage induced by UV and cancer-causing compounds in HCC [68]. In addition, restoring ARID2 expression in hepatoma cells suppressed cell growth and tumor progression in mice while ARID2 inhibition resulted in upregulation of cell cycle proteins such as cyclin D1 and cyclin E1, suggesting a tumor suppressive role for ARID2 in HCC [69].

## Histone deacetylation and HCC

Besides DNA methylation, ncRNAs and chromatin remodelers, histone modifications comprise another group of epigenetic mechanisms that play important roles in regulating gene expression and changes in chromatin structure. DNA is packed into chromatin with the help of histone protein octamers, and the amino acid residues on the histone tails that protrude from these nucleosome cores are subjected to various post-translational modifications, which includes acetylation, methylation, phosphorylation and ubiquitination [70]. These modifications affect the affinity of DNA binding to histones and are thus important for the regulation of gene transcription and expression [71]. For the purpose of this review, we chose to focus our discussion on histone deacetylases (HDACs) due to the many successful preclinical and clinical efficacies of using HDAC inhibitors in cancer. Readers can refer to other reviews for more in depth discussion on histone modifications and their implications in cancer [5, 72, 73].

Histone acetylation is a process that is regulated by two groups of enzymes with opposing functions: histone acetyltransferases (HATs), which add acetyl moieties to lysine residues, and HDACs that catalyse the removal of these acetyl groups [74]. Acetylation removes the positive charge of lysine residues, weakening the interaction of histones with negatively-charged DNA, which leads to a transcriptionally active chromatin state. In contrast, the removal of acetyl groups promotes a closed chromatin state by maintaining the strong interaction of DNA with positively-charged histones [75]. The dynamic balance between HAT and HDAC activity is critical in maintaining normal gene expression and this balance is often lost in various diseases including HCC [76].

The dysregulated expression of HDACs in HCC have been reported in several studies. Wu et al. discovered that overexpression of HDAC3 was an independent prognostic factor of tumor recurrence after liver transplantation in HBV-associated HCC patients [77]. HDAC3 also appeared to have functional roles in promoting tumor cell proliferation and invasion in vitro, indicating the potential for HDAC3 to serve as a biomarker and therapeutic target for HBV-associated HCC [77].

Besides, HDAC1 and HDAC2 have also been found to be upregulated in a cohort of Southeast Asian HCC patients, and are correlated with increased mortality [78]. The authors further demonstrated that inhibition of HDAC1/2 was able to suppress proliferation and induce tumor cell death in several HCC cell lines. Mechanistically, Yang et al. observed that upregulation of HDAC1 and HDAC2 suppresses the expression of a key metabolic enzyme in glucose metabolism, fructose-1,6-bisphosphatase (FBP1), resulting in increased lactate production in liver cancer cells [79]. The authors also showed that restoring FBP1 expression via HDAC inhibition was able to inhibit tumor cell growth in vitro and in vivo. In addition, HDAC2 knockdown transcriptomic studies revealed that HDAC2 dysregulation contributes to HCC pathogenesis by modulating expression of genes involved in apoptosis, cell cycle and lipid metabolism [80].

## Epigenetic therapies in HCC

As various epigenetic mechanisms have been found to play significant roles in contributing to HCC pathogenesis, they have also become interesting/promising targets for cancer therapy. Here, we review the current development and progress of epigenetic therapies targeted against HCC, focusing on inhibitors of DNA methylation and histone acetylation as well as miRNA-based therapies. A summary of these HCC-specific epigenetic drugs and their clinical status can be found in Table 3.

**Table 3:** Epigenetic drugs used in liver cancer-related clinical trials

Drug	Target/MOA	Clinical status for HCC	Findings/results	Ref
Azacytidine	Inhibits DNMT (acts as cytidine analogue)	Pre-clinical	Induce differentiation as a form of epigenetic reconditioning to sensitise tumor cells to sorafenib	[81]
Decitabine + chemo- or immunotherapy	Inhibits DNMT (acts as cytidine analogue)	Phase I/II (NCT01799083)	Re-sensitise tumor cells to sorafenib; effective and safe at low doses alone and in combination with chemo- or adoptive immunotherapy	[82–84]
Guadecitabine (SGI-110) + sorafenib + oxaliplatin	Inhibits DNMT (dinucleotide of deoxyguanosine and decitabine)	Phase II (NCT01752933)	Suppress tumor growth and progression, induce re-expression of silenced TSGs, alone or in combination with sorafenib; pre-treatment potentiates anti-tumor effects of oxaliplatin	[86–90]
Panobinostat	HDAC	Pre-clinical	Inhibit proliferation, induce alternative apoptosis pathways, promote differentiation and less invasive phenotype, mediate anti-angiogenic effects and cancer metabolism	[79, 91–93]
Belinostat (PXD-101)	HDAC	Phase I/II (NCT00321594)	45% patients achieved stable disease; HR23B identified as response biomarker	[94, 95]
Resminostat + sorafenib	HDAC	Phase I/II (NCT00943449)	Induce more epithelial phenotype and potentiate sorafenib-induced cell death; combination treatment with sorafenib prolonged TTP and OS in HCC patients	[96–98]
CUDC-101	Inhibits HDAC, EGFR, HER2	Phase Ib (NCT01171924)	Block tumor growth in vitro and in vivo; acceptable safety profile in patients	[99, 100]
Anti-miR-221	Inhibits miR-221 (AMO)	Pre-clinical	Inhibit tumorigenic effects of miR-221; miRNA sponges sustain miR-221 depletion and induce apoptosis	[101, 102]
Miravirsen	Inhibits miR-122 (LNA-modified AMO)	Phase IIa (NCT01200420)	Highly specific for miR-122; sustained suppression of HCV infection with high genetic barrier to resistance in patients; no long-term safety issues or AE	[107–110]
miR-185 mimic	Exogenous miR-185 oligonucleotide	Pre-clinical	Suppress tumor cell proliferation and invasion; targets DNMT1/PTEN/Akt axis	[111]

*DNMT* DNA methyltransferase, *HDAC* histone deacetylase, *EGFR* epidermal growth factor receptor, *HER2* human epidermal growth factor receptor 2, *AMO* anti-miRNA oligonucleotides, *LNA* locked nucleic acid, *TSG* tumor suppressor gene, *TTP* time to progression, *OS* overall survival, *AE* adverse events

### DNA methylation inhibitors

Small molecule inhibitors of DNA methyltransferase (DNMT) were the earliest group of epigenetic drugs to be studied as an alternative approach to cancer treatment. In fact, azacytidine was the first epigenetic drug to be approved by the FDA in 2004 for the treatment of myelodysplastic syndrome (MDS). Since then, various DNMT inhibitors have been developed and tested against multiple cancer indications, including HCC, with promising preclinical results. Inhibitors of DNA methylation can be divided into two broad classes based on their mechanism—nucleoside analogues and non-nucleoside compounds. Many of the first generation DNMT inhibitors such as azacytidine (5-azacytidine) and decitabine (5-aza-2′-deoxycytidine) function as analogues of cytosine, and their incorporation into DNA prevents methylation by DNMTs.

Besides de-repressing silenced tumor suppressor genes, DNMT inhibitors can have other anti-cancer effects. A recent study by Gailhouste et al. demonstrated the ability of azacytidine in inducing hepatic cancer cell differentiation [81]. Using non-cytotoxic doses of azacytidine to achieve “epigenetic reconditioning”, the authors observed reduced tumor formation ability in mouse xenograft models and reported that azacytidine treatment sensitised the tumor cells to sorafenib, notably by converting the more drug-resistant liver progenitor-like cancer cells into mature hepatocytes. This study highlights the potential role of pre-treating liver cancer cells with DNMT inhibitors to recondition and prime these cells for more effective killing by other targeted or chemotherapeutic agents.

The use of DNMT inhibitors in sensitising HCC cells to sorafenib has also been reported in other studies. Liu et al. identified a possible mechanism of sorafenib resistance in HCC via the upregulation of NFκB/PDL1/STAT3/DNMT1 axis, leading to hypermethylation and silencing of tumor suppressor Cadherin 1 (CDH1) [82]. They showed that decitabine was able to re-sensitise resistant tumor cells to sorafenib and decreased colony formation ability. Due to the promising preclinical data, decitabine has been tested in various clinical trials for different indications. To mitigate the adverse events that accompany the usual dose of decitabine for treating solid tumors, two phase I/II trials have been conducted to examine the efficacy of using low-dose decitabine, alone or in combination with chemotherapy or adoptive immunotherapy [83, 84]. These trials demonstrated acceptable safety and toxicity profiles of low-dose decitabine in HCC, and the study by Fan et al. suggested the promising role of low-dose decitabine-based chemo-or immunotherapy in the treatment of cancer [84].

Despite the promising preclinical data, these DNMT inhibitors generally have short-half-lives due to metabolic inactivation by cytidine deaminase (highly expressed in the liver), which significantly reduces their efficacy in vivo [85]. Hence to overcome these limitations, second generation DNMT inhibitors have been developed, including guadecitabine (SGI-110). This compound was designed as a dinucleotide linking a deoxyguanosine with decitabine to enhance the stability of nucleoside analogues against degradation by cytidine deaminase [86]. In preclinical models, SGI-110 was able to suppress HCC progression and induce re-expression of tumor suppressor genes when used alone or in combination with sorafenib [87, 88]. This dinucleotide inhibitor recently completed phase II trials for advanced HCC patients who are refractory to sorafenib treatment and the results of its clinical efficacy are currently being awaited [89]. The ability of guadecitabine to prime HCC cells to other treatments has also been shown in a preclinical study by Kuang et al. [90]. Using HCC cell lines and mouse xenograft models, the authors demonstrated that pre-treatment with low-dose guadecitabine primed the tumor cells to oxaliplatin, resulting in enhanced cytotoxic and antiproliferative effects on tumor growth when compared to oxaliplatin treatment alone.

### Histone deacetylation inhibitors

In many cancers, aberrant histone deacetylation, which results in deregulated gene silencing of important tumor suppressors, has emerged as a promising target for therapy. While several HDAC inhibitors have been approved by the FDA for treatment of haematological malignancies, the efficacy of these compounds in liver cancer is still being investigated, either pre-clinically or clinically.

Panobinostat is a pan-HDAC inhibitor that has been found to inhibit tumor cell proliferation, induce alternative pathways of apoptosis [91] and promote a more differentiated and less invasive phenotype in HCC cells [92]. In addition, alternative mechanisms of panobinostat activity have recently been discovered, including its ability to mediate anti-angiogenic effects via connective tissue growth factor (CTGF) pathway [93]. Moreover, panobinostat can affect cancer metabolism and tumor growth by restoring the expression of a key gluconeogenesis enzyme, fructose-1,6-bisphosphatase (FBP1), which is silenced by histone deacetylation in many cancers [79]. The clinical efficacy of panobinostat has been evaluated in several phase I trials as a combination therapy with sorafenib; however, no promising results were observed, and dose-limiting toxicities led to the termination of one such trial.

Another pan-HDAC inhibitor that has been shown to have antiproliferative and proapoptotic effects in HCC is belinostat [94]. In a phase I/II clinical trial that evaluated belinostat for the treatment of advanced unresectable HCC, the median PFS and OS were 2.64 and 6.6 months respectively, with 45.2% of patients achieving stable disease [95]. Interestingly, Yeo et al. also identified HR23B, a carrier protein involved in delivering ubiquitinated proteins to the proteasome, as a potentially useful biomarker for predicting response to HDAC inhibitors. Recently, a phase I/II trial of resminostat was conducted on advanced HCC patients who progressed with sorafenib to compare the efficacy of resminostat as a single treatment and in combination with sorafenib. The results from the study indicated poor efficacy of resminostat alone but showed that the HDAC inhibitor could restore sensitivity to sorafenib [96]. The median time to progression (TTP) and overall survival (OS) for resminostat monotherapy were 1.8 and 4.1 months respectively, whereas the combination of resminostat and sorafenib prolonged the TTP and OS to 6.5 and 8.0 months respectively [96].

Mechanistically, Fu et al. demonstrated that the cytotoxic effects of resminostat in HCC cell lines and patient-derived primary cells were dependent on activation of the mitochondrial apoptotic pathway [97]. In addition, the synergistic effects of resminostat and sorafenib were observed when addition of low dose resminostat enhanced sorafenib-induced mitochondrial apoptosis pathway [97]. These mechanisms were further investigated by Soukupova et al. recently, in which the authors postulated that changes in the epithelial-mesenchymal phenotype could be a sensitisation mechanism of HDAC inhibition [98]. They found that treatment of hepatic cancer cells with resminostat induced a more epithelial phenotype with less invasive and stem-like properties, which sensitised the cells to sorafenib-induced apoptosis [98].

As HDAC inhibitors have been shown to synergise or potentiate the effects of other anti-cancer therapies, there is great promise in using these drugs for combination therapy. Lai et al. adopted a similar approach but instead of using multiple drugs in combination, they designed a single small molecule inhibitor to work against various molecular targets [99]. CUDC-101 is novel compound that simultaneously targets HDAC along with epidermal growth factor receptor (EGFR) and human epidermal growth factor receptor 2 (HER2) [99]. The authors reported that CUDC-101 could effectively inhibit tumor cell growth and proliferation in vitro and in vivo, highlighting the potential of multi-targeted inhibitors as a new paradigm for the treatment of heterogenous and drug-resistant tumors. Subsequently, the safety of CUDC-101 was assessed in a phase Ib (expansion) study, and it was found to be well-tolerated in patients with advanced solid tumors including liver cancer, with early evidence of antitumor activity [100].

### RNA-based epigenetic therapy

Various microRNAs (miRNAs) which are involved in regulating or being regulated by epigenetic processes have also been implicated in process of hepatic tumorigenesis, making them rational targets for miRNA-based strategies in epigenetic therapy. Several studies have established the therapeutic value of targeting aberrantly upregulated miRNAs in cancer, and miRNA inhibition can be achieved via several methods, including miRNA antisense oligonucleotides (AMOs) and miRNA sponges. Callegari et al. demonstrated that delivery of anti-miR-221 AMOs could reverse the tumorigenic effects of miR-221 in a transgenic mouse model [101]. To prolong the miRNA inhibition effects, Moshiri et al. devised an alternative approach using vector-encoded “miRNA sponges” [102]. These sponges contain several miR-221 antisense binding sites that can sequester miR-221 competitively, thus preventing its function. From the results of the study, miR-221 sponges were able to deplete endogenous miR-221 and its target genes as well as promote apoptosis in HCC cells [102]. While no tests have been conducted using miR-221 sponges in vivo yet, it would be interesting to examine the safety and efficacy of these sponges in a more clinically relevant setting for the treatment of HCC.

Another promising miRNA target for therapy is miR-122. In contrast to miR-221, miR-122 is found to act as a tumor suppressor in the liver [103, 104]. However, miR-122 levels do not always decrease in all cases of HCC and is dependent on the disease aetiology. For example, miR-122 has been found to be highly expressed in HCV-related HCC [105, 106], making it a relevant target for antiviral therapies aimed at impeding liver disease progression. Miravirsen is a locked nucleic acid (LNA)-modified antisense oligonucleotide that inhibits miR-122 by forming highly stable heteroduplexes with it [107]. In preclinical studies, miravirsen was able to sustain the suppression of HCV RNA levels in chronic HCV-infected chimpanzees, without evidence of resistance-conferring mutations developing in miR-122, and this was also observed in results from a phase IIa clinical trial [107, 108]. No dose-limiting adverse events were reported and miravirsen treatment was additionally found to reduce serum cholesterol levels, indicating possible relevance for use in fatty liver disease intervention [107]. The safety and efficacy of miravirsen were further evaluated by van der Ree et al. [109]. They reported that the 27 patients treated with miravirsen displayed no long-term safety issues after 35 months of follow-up, and these patients did not develop further liver diseases including HCV-related HCC [109]. In addition, miravirsen was shown to be highly specific for miR-122 as plasma levels of other miRNAs in patients treated with the inhibitor were unaffected [110].

Aside from miRNA inhibition, miRNAs can also be modulated by miRNA mimics, especially for tumor suppressive miRNAs that are often downregulated or suppressed in HCC. One such example is miR-185. Qadir et al. reported that modulating miR-185 expression using miRNA mimic oligonucleotides was able to suppress HCC cell growth and invasion [111]. They demonstrated that the tumor suppressive effects of miR-185 occurred via DNMT1 as overexpression of miR-185 depleted DNMT1, resulting in PTEN induction and subsequent inhibition of Akt. These promising preclinical data led the authors to propose miR-185 reactivation as a novel strategy for HCC treatment [111].

## Conclusions

The liver cancer epigenome is highly complex and is adapted to changing environmental and developmental cues. While most of these epigenetic studies identified potential targets for therapeutic intervention in liver cancer, most studies lacked in-depth validations. The diversity of epigenetic alterations in different subsets of liver cancer is still poorly understood. Most studies focused on promoter hyper/hypo-methylation with lesser studies focusing on non-promoter or global histone modifications, non-coding RNAs, chromatin architecture and their integrated analysis. Since liver cancer is a highly heterogeneous disease, detailed analysis of epigenetic changes contributed by specific cell type in the bulk liver tumor should be carried out. Integration of laser capture microdissection, single cell analysis and flow cytometry cell sorting technologies could be incorporated with genome-wide studies in the future. With better understanding of how various epigenetic modifiers interact with one another to alter and maintain the epigenomic landscape, one can develop more clinically effective targeted epigenomic therapy for liver cancer.
